# Peroxidase Profiling Reveals Genetic Linkage between Peroxidase Gene Clusters and Basal Host and Non-Host Resistance to Rusts and Mildew in Barley

**DOI:** 10.1371/journal.pone.0010495

**Published:** 2010-08-02

**Authors:** Ana M. González, Thierry C. Marcel, Zuzana Kohutova, Piet Stam, C. Gerard van der Linden, Rients E. Niks

**Affiliations:** Laboratory of Plant Breeding, Wageningen University and Research Center (WUR), Wageningen, The Netherlands; Umeå Plant Science Centre, Sweden

## Abstract

**Background:**

Higher plants possess a large multigene family encoding secreted class III peroxidase (*Prx*) proteins. Peroxidases appear to be associated with plant disease resistance based on observations of induction during disease challenge and the presence or absence of isozymes in resistant *vs* susceptible varieties. Despite these associations, there is no evidence that allelic variation of peroxidases directly determines levels of disease resistance.

**Methodology/Principal Findings:**

The current study introduces a new strategy called *Prx*-Profiling. We showed that with this strategy a large number of peroxidase genes can be mapped on the barley genome. In order to obtain an estimate of the total number of *Prx* clusters we followed a re-sampling procedure, which indicated that the barley genome contains about 40 peroxidase gene clusters. We examined the association between the *Prxs* mapped and the QTLs for resistance of barley to homologous and heterologous rusts, and to the barley powdery mildew fungus. We report that 61% of the QTLs for partial resistance to *P. hordei*, 61% of the QTLs for resistance to *B. graminis* and 47% of the QTLs for non-host resistance to other *Puccinia* species co-localize with *Prx* based markers.

**Conclusions/Significance:**

We conclude that *Prx*-Profiling was effective in finding the genetic location of *Prx* genes on the barley genome. The finding that QTLs for basal resistance to rusts and powdery mildew fungi tend to co-locate with *Prx* clusters provides a base for exploring the functional role of *Prx*-related genes in determining natural differences in levels of basal resistance.

## Introduction

Class III plant peroxidases (EC 1.11.1.7; *Prxs*) are enzymes that catalyze oxidoreduction between H_2_O_2_ and various reductants and are involved in a broad range of physiological processes, including plant defense [1]. Because they are induced by fungi [2], bacteria [3], [4], viruses [5] and viroids [6], they are considered as pathogenesis-related (PR) proteins, belonging to the PR-protein 9 subfamily [7]. One of the roles of *Prxs* in plant defense is the reinforcement of cell wall physical barriers and lignification [8]–[10].

There is evidence that defense-related genes like those encoding peroxidase (PR-9), superoxide dismutase and thaumatin-like protein (PR-5) are potential candidates to explain quantitative resistance to plant pathogens [11], [12]. Indeed, in earlier studies we identified six *Prx* genes to map within 1cM from markers associated with several quantitative trait loci (QTLs) contributing to basal resistance to barley leaf rust (*Puccinia hordei* Otth; [13]) and non-host resistance to several unadapted rust fungi [14]. Others have shown that the barley *HvPrx7* peroxidase mRNA accumulates in response to the powdery mildew fungus (*Blumeria graminis* f.sp. *hordei*) in barley leaves [15] and in roots as reaction to *Pyrenophora graminea* [16]. *HvPrx7* was also implicated as a susceptibility factor in barley, enhancing successful haustorium formation by the powdery mildew fungus [17]. Another peroxidase of barley, *HvPrx8* is pathogen-induced at the mRNA as well as protein level [15]. Transient overexpression of *HvPrx40* enhanced the resistance of wheat (*Triticum aestivum*) and barley against wheat and barley powdery mildew, respectively [18]. The fact that basal host and non-host resistance to rusts and powdery mildew fungi are mediated by the formation of cell wall appositions (papillae) [19]–[21] also supports the qualification of *Prxs* as candidate genes determining the level of resistance.

The various reports associating *Prx* activity to defense and stress responses justify an attempt to determine the number of *Prx* gene clusters in barley, and the degree of association of those clusters with QTLs for resistance against rust and mildew. Extensive work on various mapping populations and with various cereal and grass rust species and barley powdery mildew has resulted in a large number of mapped QTLs that contribute to quantitative resistance in barley [13], [14], [22], [23].

We followed the Motif-directed Profiling approach [24], [25] that targets conserved motifs in functional domains of gene family members, thus sampling genetic variation in and around members of a particular gene family. The nucleotide-binding site (NBS) Profiling technique is an example of Motif-directed Profiling that targets resistance genes (*R*-genes) and *R*-gene analogues (RGAs) by using degenerate primers that are homologous to conserved sequences in the NBS domain of the NBS-LRR (NBS leucine-rich repeat) class of *R*-genes [26].

The Motif-directed Profiling approach can be applied to any gene family that has multiple members (at least 30–40), and has one or more conserved sequence motif(s) to allow selective binding of a (degenerate) primer to many gene family members. *Prx* genes fulfill both requirements. Analyses of rice (138 peroxidase genes and 14 pseudogenes; [27]), *Brachypodium distachyon* (173 peroxidase genes; [28]) and Arabidopsis (73 peroxidase genes; [29]) genomes suggest a large number of *Prx* genes to be found in plants. They tend to occur in clusters in the genome [27]. In the cereal crop barley a similar level of complexity of class III peroxidases appears as in rice and Arabidopsis, with a total of 124 unigenes presently known (PeroxiBase, August 2009, http://peroxibase.isb-sib.ch/). Class III plant peroxidases also fulfill the condition of containing several conserved motifs [27], [29], [30], [31].

Here we report the effective use of Peroxidase Profiling to map new dedicated markers homologous to *Prx* genes in barley. Identification and mapping of *Prx* genes in barley provides new markers for genetic mapping and for the discovery of sequences that may characterize resistance QTLs. Our aims were to: (1) assess the efficiency of the *Prx* Profiling method to develop *Prx*-based markers in a segregating barley progeny; (2) determine the overall genomic organization of peroxidases in barley and predict the total number of *Prx* clusters in barley; (3) investigate whether the QTLs for resistance to barley rust, barley mildew and heterologous rust fungi tend to be located in *Prx* clusters.

## Results

### *Prx* Profiling and Level of Polymorphism

First we examined all 36 combinations of 12 primers with three restriction enzymes (*MseI*, *RsaI*, and *AluI*) on 11 barley genotypes (parents of mapping populations), including Vada, L94 and SusPtrit, to select combinations with optimal number of polymorphic bands. Once the optimal primer-enzyme combinations were identified, *Prx* Profiling was applied on both mapping populations (L94 × Vada, Vada × SusPtrit).

Twelve degenerate primer-enzyme combinations were used for mapping: PERO1.*MseI*, PERO2.*MseI*, PERO3.*MseI*, PERO4.*MseI*, PERO5.*MseI*, PERO6.*MseI*, PERO1.*RsaI*, PERO3.*RsaI*, PERO4.*RsaI*, PERO5.*RsaI* PERO1.*AluI*, PERO2.*AluI*. These combinations produced 1292 bands, 185 of which were polymorphic: 93 and 92 polymorphic bands in L94×Vada and Vada×SusPtrit crosses, respectively (Table 1; Figure S1). Mean polymorphism rates detected using *MseI*, *RsaI* and *AluI* as restriction enzymes were 14%, 13% and 18%, respectively. Mean number of polymorphic bands per enzyme-primer combination was 15.4, ranging from 4 (PERO3.*MseI*) to 30 (PERO2.*MseI*) polymorphic bands. The populations did not differ from each other in their level of polymorphism (14.2 and 14.4%).

**Table 1:** Level of polymorphism of the 12 primer-enzyme combinations used for *Prx* profiling of barley RIL populations.

Primer.Enzyme combination	Amplified bands	Polymorphic bands	
		SusPtrit×V	L94×V
PERO1.*MseI*	74	9	10
PERO2.*MseI*	128	21	7
PERO3.*MseI*	70	0	4
PERO4.*MseI*	155	9	12
PERO5.*MseI*	156	9	8
PERO6.*MseI*	125	2	8
PERO1.*RsaI*	86	7	10
PERO3.*RsaI*	63	4	4
PERO4.*RsaI*	100	5	6
PERO5.*RsaI*	119	6	5
PERO1.*AluI*	105	12	11
PERO2.*AluI*	111	8	8
Total	1292	92	93

The FHDCFV-derived primers produced fewer amplified bands (637 FHDCFV-derived bands *vs* 655 VSCADI-derived bands), but more polymorphic bands for all primer/enzyme combinations (115 FHDCFV-derived bands *vs* 70 VSCADI-derived bands)

Primers targeting the same conserved motif (FHDCFV or VSCADI) but at slightly different positions and with slightly different nucleotide compositions produced different DNA fingerprints, indicating that different subsets of *Prx*-genes were targeted by these primers. The primers can be used in other plant species as well since the motifs targeted by the degenerated primers are known to be highly conserved in the plant kingdom [31]. DNA fingerprints with some of the primers presented here were also successfully produced for potato and *Miscanthus* (work in progress).

### Genetic Mapping

**Figure 1:**
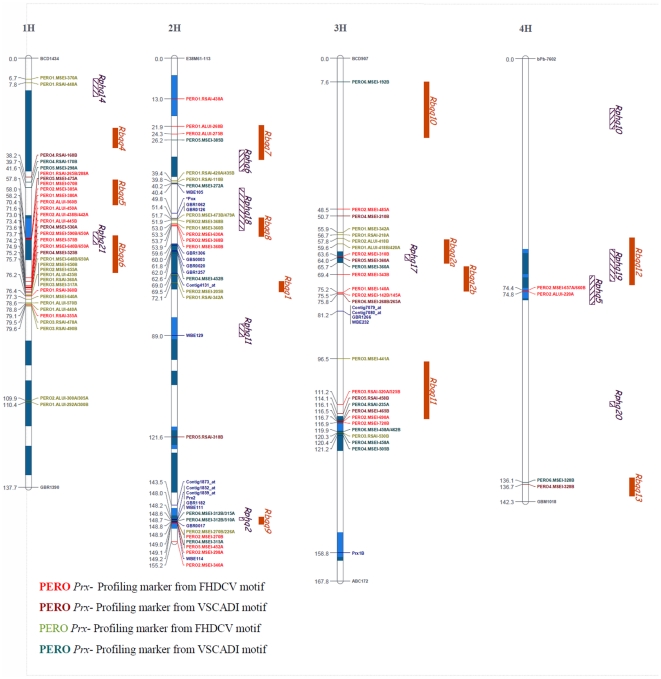
Location of 200 *Prx*-targeted markers on a high–density integrated map of barley, linkage groups 1H to 4H [22], to be continued in Figure 2. The QTLs were originally mapped in several individual barley linkage maps [13], [14]. Lengths of QTL boxes correspond to the LOD-1 support intervals (from the peak marker) on the basis of results of restricted (r) MQM. Numbers on the left side show the distance in centiMorgans (according to Kosambi) from the top of each chromosome. The red markers correspond to *Prx* markers mapped in Vada×SusPtrit progenies, the green markers correspond to *Prx* markers mapped in L94×Vada progenies, and the blue markers correspond to *Prx* based molecular markers that were available from different sources, such as ESTs. In the cases of different markers matching the same position, the markers are adjacent on the same line. The black markers correspond to the first and the last marker of the linkage group. Different colours of blue inside the chromosome bars correspond to QTL for basal host and non-host resistances that overlapped.

**Figure 2:**
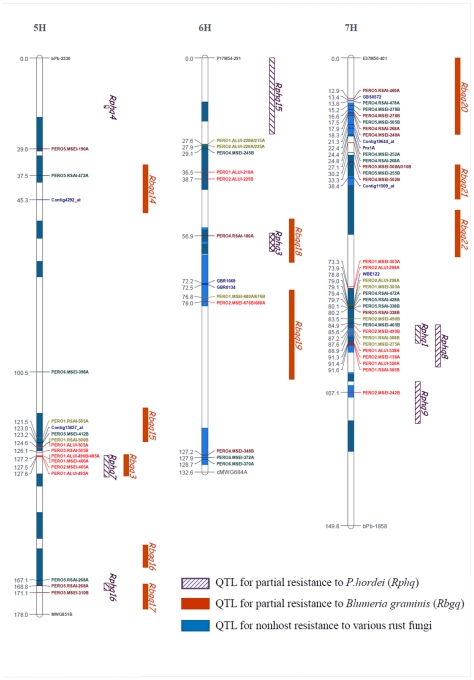
Location of 200 *Prx*-targeted markers on a high-density integrated map of barley, linkage groups 5H to 7H [22], continued from Figure 1. The QTLs were originally mapped in several individual barley linkage maps [13], [14]. Lengths of QTL boxes correspond to the LOD-1 support intervals (from the peak marker) on the basis of results of restricted (r) MQM. Numbers on the left side show the distance in centiMorgans (according to Kosambi) from the top of each chromosome. The red markers correspond to *Prx* markers mapped in Vada x SusPtrit progenies, the green markers correspond to Prx markers mapped in L94 x Vada progenies, and the blue markers correspond to *Prx* based molecular markers that were available from different sources, such as ESTs. In the cases of different markers matching the same position, the markers are adjacent on the same line. The black markers correspond to the first and the last marker of the linkage group. Different colours of blue inside the chromosome bars correspond to QTL for basal host and non-host resistances that overlapped.

Nine of the polymorphic bands in the L94×Vada and eight in the Vada×SusPtrit RILs were excluded because they could not be mapped to linkage groups without changing marker order and genetic distances. Finally, 168 polymorphic bands (84 in each population) were mapped and placed on an integrated map of barley [22] (Table S1). These 168 *Prx* Profiling markers (identified by the label PERO in the marker name, e.g. Figures 1 and 2). were added to 32 *Prx* markers that were mapped previously [13], [22], and that were considered to be Defense Gene Homologues (DGH). This made a total of 200 *Prx*-based markers. These were not homogeneously distributed among the seven chromosomes and tended to map in clusters (Figures 1 and 2). Both populations showed a similar distribution of the markers, with the lowest number of *Prx* Profiling markers for chromosome 4H (4 PERO-markers for Vada×SusPtrit and 1 for L94×Vada). Chromosomes 1H and 7H had the highest number of markers (with 21 PERO-markers on 1H and 16 PERO-markers on 7H for Vada×SusPtrit and 20 PERO-markers on 1H and 15 PERO-markers on 7H for L94×Vada).

Both populations shared Vada as parental line. Out of the 168 mapped *Prx* Profiling markers, only 12 were unambiguously common to both populations (Table S1): seven amplified from Vada, and five amplified from L94 and SusPtrit. These markers were obtained with the same primer/enzyme combination, and had the same fragment size and the same or almost the same marker position on the integrated map. In addition to these common markers between populations, within each population there were 50 bands (25 pairs: 12 in Vada×SusPtrit and 13 in L94×Vada: Table S1) that may represent alternative alleles of the same gene. They were produced by the same primer/enzyme combination, generated a band of different size in both parents and mapped at the same location. These markers could be paralogous genes in close proximity of each other or the same genes. These markers are indicated separately in Figures 1 and 2, and are counted as distinct markers.

### Sequence Analysis of the *Prx* Bands

In order to verify whether the amplified fragments represent *Prx* genes/pseudogenes, a sample of bands was excised from polyacrylamide gels and sequenced with both specific and adapter primers. For only 35 (57%) of 61 excised bands we obtained reliable sequences (Tables S2 and S3). The relatively low rate of successful sequencing reactions is not surprising since the excision of single bands from polyacrylamide gels is not a trivial task. The most likely cause for bad sequence quality is that more than a single product is excised from the gel and sequenced, especially if the target band migrates very close to other bands of nearly similar sizes.

Of the 35 bands from which we obtained a useful sequence (Tables S2 and S3), ten were monomorphic in the populations tested, one was polymorphic in L94×Vada but unmapped, and 24 were polymorphic and mapped in one of the two barley populations (Table 2).

**Table 2:** Details of sequences obtained from peroxidase profiling amplified bands and their homology to known peroxidase sequences.

Primer	Motif^1^	Markers^2^	Seq.^3^	Marker seq.^4^	*Prx* hits^5^ (*E* <1)	*Prx* hits^5^ (*E* <10^−5^)	Barley *Prx* hits^5^ (*E* <1)
PERO1	FHDCFV	55	17	16	15	13	15
PERO2	FHDCFV	42	6	6	6	5	5
PERO3	FHDCFV	10	0	-	-	-	-
PERO4	VSCADI	29	7	1	7	2	3
PERO5	VSCADI	22	2	0	1	0	0
PERO6	VSCADI	10	3	1	1	0	0
*Total*		*168*	*35*	*24*	*30*	*20*	*23*

1 Conserved peroxidase motif on which the *Prx* primer was developed;

2 Number of *Prx* profiling markers mapped on the barley integrated map;

3 Number of sequences obtained;

4 Number of the sequences obtained that correspond to a mapped marker;

5 Number of sequences having a BLASTx hit in the PeroxiBase (http://peroxibase.isb-sib.ch/) with the corresponding *E value*.

Out of the 35 successfully sequenced amplified fragments, 20 (57%) had strong homology (*E* value<10^−5^) to peroxidase protein sequences in PeroxiBase and NCBI after BLASTX analysis (Tables 2 and S2). Amplified fragments based on a FHDCFV-containing motif primer (PERO1, PERO2) had much more frequently a strong homology (*E* value<10^−5^) to peroxidase protein sequences in PeroxiBase and NCBI after BLASTX analysis (18 out of 23) than the ones based on a VSCADI-containing motif primer (PERO4, PERO5, PERO6) (Table S2). The finding that a large proportion of the excised bands (30 out of 35) had a BLASTX hit in PeroxiBase suggests that the majority of PERO-markers indeed are located in *Prx* gene sequences.

### Prediction of the Number of *Prx* Gene Clusters on the Barley Genome

With the re-sampling procedure described in Material and Methods, we estimated the total number of *Prx* clusters in the barley genome.

We arbitrarily considered *Prx* based markers as to belong to the same cluster when the largest distance between adjacent markers did not exceed 5 cM. This choice of 5 cM is the average size of BINs on the integrated map. Moreover, 5 cM is near the average distance between consecutive markers on the framework map (containing only markers common to two or more populations).

On the basis of the map positions of the *Prx* based markers (Figures 1 and 2) we found a total of 40 clusters, varying from “clusters” of a single *Prx* (14 clusters) to one cluster containing 26 *Prx* Profiling markers (Figure S2).

**Figure 3:**
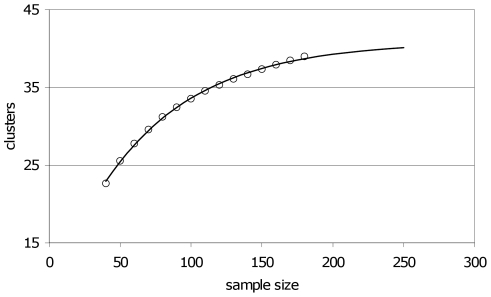
Results of the re-sampling procedure. Each data point represents the mean of 50,000 re-sampling runs. Shown is the relation between sample size and average number of realized clusters in the sample. Curve: exponential curve fitted to the data; the horizontal asymptote equals 40.9. See text for details.

The re-sampling procedure resulted in a curve that approached an asymptotic value of about 41 (Figure 3), indicating that the clusters we have found so far most likely cover over 95% of the total number of *Prx* gene clusters in barley.

Since there is no *a priori* theoretical basis to fit a saturation curve of a certain type, we decided to try two types, *i.e.* the exponential and (rectangular) hyperbolic ones. Though both types of curves fitted almost equally well (in both cases more than 98% variance explained by regression), they had clearly different horizontal asymptotes. Therefore, we investigated the general predictive power of these two types of curves in the following way.

We took a random sample of given size from the 200 *Prx* markers and – for the current purpose – considered this to be the true constellation of markers and clusters. Then, the re-sampling procedure as described above was applied to this supposedly true constellation. The predicted upper limits were determined by fitting two alternative curves (exponential and hyperbolic). This procedure was applied to a number of samples of various sizes that were considered as ‘true constellations’. It turned out that fitted exponential curves predicted the number of clusters slightly better than hyperbolic curves. For that reason we eventually applied exponential curve fitting to our data.

### Association of *Prx* Based Markers with QTLs for Resistance

The possible association between *Prx* based markers and several types of resistance loci was investigated in this study. The integrated map was divided into 217 BINs of approximately 5 cM. A BIN can either be occupied by one or more *Prx* based marker(s), or by one or more QTL peak marker(s), or by both (or by neither of them). This enabled the construction of 2×2 contingency tables that allow tests of independence regarding the occupancy by *Prx* based markers and QTL peak markers (cf. [13], [14]). The 200 *Prx* based markers occupied only 63 BINs due to strong clustering (Table 3).

**Table 3:** Chi-square values on the probability of independent distribution of *Prx* based markers with barley QTLs for partial resistance to *Puccinia hordei* (QTLph), to *Blumeria graminis* (QTLbg), nonhost resistance to heterologous cereal and grass rusts (QTLnh), days to heading (QTLdh), diastatic power (QTLdp), plant height (QTLplh), kernel weight (QTLkw), test weight (QTLtw) and yield (QTLyi).

	*Prx*	QTLph^1^	QTLbg	QTLnh	QTLdh	QTLdp	QTLplh	QTLkw	QTLtw	QTLyi
**Marker no.** 2	200	19	23	63	52	15	31	13	18	24
**BIN no.** 3	63	18	23	47	39	9	28	11	13	23
**O (E)** 4		11 (5.2)	14 (6.7)	22 (13.4)	15 (11.5)	3 (2.6)	12 (8.3)	5 (3.2)	5 (3.8)	9 (6.8)
**χ^2^** 5		9.9 ^*^	12.6^**^	9.9^*^	1.8	0.2	2.8	1.4	0.6	1.1

1 Mapping data were obtained from previous works [13], [14], [22], [32], [33], [34], [35].

2 The number of markers or QTL peak markers mapped on the integrated map of barley.

3 The number of barley BINs (5 cM) occupied by the (peak) markers for the respective class of QTLs or markers.

4 Number of BINs observed to be co-occupied by a QTL peak marker and a *Prx*-targeted marker/other marker (the expected number of co-occupied BINs is in brackets).

5 Chi-square values in bold indicate the rejection of independent distribution with a probability *P*<0.001 (with 1 d.f., *P* = 0.001 for χ^2^ = 10.83) or *P*<0.05 (with 1 d.f., *P* = 0.05 for χ^2^ = 3.84).

We compared the position of *Prx* based markers on the integrated map with the position of QTLs in five of the populations composing the integrated map: QTLs for basal resistance to barley leaf rust (19 QTLs), powdery mildew (23 QTLs) and non-host resistance to seven heterologous rusts (63 QTLs) mapped on the integrated barley map. We also compared the position of the *Prx* based markers with the position of QTLs for morphological and agronomic traits mapped on various barley mapping populations: days to heading (52 QTLs), diastatic power (15 QTLs), plant height (31 QTLs), kernel weight (13 QTLs), test weight (18 QTLs) and yield (24 QTLs). QTL position data sets were downloaded from the publicly available GrainGenes 2.0 database (http://wheat.pw.usda.gov/GG2/index.shtml).

Nineteen QTLs for resistance to *P. hordei* [13], [14], [32], [33] were placed on the integrated map and occupied 18 BINs (Table S4). The chi-square test indicated a significant association between the distribution of *Prx* based markers and QTLs for basal resistance to *P. hordei* (*P*>0.05) (Table 3). In total, 11 BINs harbored a *Prx* based marker and a peak marker for basal resistance to barley leaf rust. The expected number of co-occupied BINs would have been 5.2 under the assumption of independent distribution of those QTLs.

The association between the distribution of *Prx* based markers and the distribution of 23 QTLs for basal resistance to *Blumeria graminis* [22], [34] (Tables 3 and S4) was even stronger (*P*>0.001), with 14 observed co-occupied BINs against 6.7 expected in case of independent distribution.

Also a significant association was found between *Prx* based markers and QTLs for non-host resistance that were reported by Niks and associates to seven species of non-adapted rust fungi ([14], [35] and unpublished QTLs by Jafary and Niks, and Alemu and Niks) (Table 3).

Seven of the BINs containing the peak marker of a resistance QTL also contain a *Prx* Profiling marker confirmed to be homologous to a known *Prx* (viz. 1H_12.2, 2H_4.2, 2H_15.1, 3H_5.2, 5H_12.1, 6H_8.1, 7H_7.2) (Table S2). In addition, some *Prx* Profiling markers are associated with more than one resistance QTL. For example, *Prx* Profiling markers in BIN 2H_15.1 are associated with QTLs for resistance against barley leaf rust, barley mildew and two heterologous rusts (*Puccinia persistens* and *P. triticina*). In total, 61% of the QTLs for partial resistance to *P. hordei*, 61% of the QTLs for resistance to *B. graminis* and 47% of the QTLs for non-host resistance to other *Puccinia* species co-localize with *Prx* based markers. Those QTLs that co-localized with *Prx* based markers did not differ from those that did not co-localize in their average percentage of explained variance of the resistance (Table S4).

Association of QTLs for resistance with *Prx* genes may be due to the occurrence of gene-rich areas rather than because of functional association. We tested whether *Prx* based markers were also associated with QTLs for days to heading (QTLdh), diastatic power (QTLdp), plant height (QTLplh), kernel weight (QTLkw), test weight (QTLtw), and yield (QTLyi) (Table 3). None of these tests indicated a significant association between the *Prx* based markers and such agronomic trait QTLs. Moreover, we used the same method to test for possible associations between the distribution of all 105 QTLs for resistance (to *P. hordei*, *B. graminis* and heterologous rusts) that occupied 70 BINs, and the distribution of four different sets of markers: 97 DGHs, 244 GBM and 34 scssr markers (EST SSRs), and 97 Bmac+Bmag (genomic SSRs) (Table 4). Thirty-two of the 97 DGH-based markers involve *Prx* genes and they occupied 15 BINs. The DGH marker loci were not significantly associated with the resistance QTLs when excluding *Prx*-DGHs (65 DGHs), but they became significantly associated when we included the 15 BINs containing *Prx*-DGHs (97 DGHs). This change from not-significant to significant association was only partly due to the effect of the increased power of the test resulting from the increased number of involved BINs from 48 to 63. Out of the 15 extra BINs containing *Prx*-DGHs, nine also contained one or more resistance QTL.

**Table 4:** Chi-square values on the probability of independent distribution of QTLs for all types of resistance (QTLres) with DGH-based markers (with and without *Prx*), and three sets of microsatellites: GBM, scssr and Bmac+Bmag markers.

	QTLres^1^	DGH (*Prx*)	DGH	GBM	scssr	Bmac+Bmag
**Marker no.** 2	105	97	65	244	34	97
**BIN no.** 3	70	63	48	133	28	66
**O (E)** 4		27 (20.3)	18(15.5)	48 (42.9)	7 (9)	21 (21.3)
**χ^2^** 5		4.6*	0.8	2.3	1.0	0.02

1 Mapping data were obtained from previous works [13], [14], [22], [32], [33], [34], [35].

2 The number of markers or QTL peak markers mapped on the integrated map of barley.

3 The number of barley BINs (5 cM) occupied by the (peak) markers for the respective class of QTLs or markers.

4 Number of BINs observed to be co-occupied by a QTL peak marker and a *Prx*-targeted marker/other marker (the expected number of co-occupied BINs is in brackets).

5 Chi-square values in bold indicate the rejection of independent distribution with a probability *P*<0.001 (with 1 d.f., *P* = 0.001 for χ^2^ = 10.83) or *P*<0.05 (with 1 d.f., *P* = 0.05 for χ^2^ = 3.84).

## Discussion

### Efficiency of *Prx* Profiling

We present here an application of the Motif-directed Profiling approach that selectively targets peroxidase genes. To our knowledge, this is the first report on the application of the gene-targeted Profiling technique described by van der Linden and associates [26] to produce markers in *Prx* genes. The presented results demonstrate the utility of this technique for *Prx* mapping. The primers developed and applied in this paper were targeted to the DNA sequence encoding the conserved FHDCFV and VSCADI amino acid sequence motifs of peroxidase proteins. These motifs are conserved in class III peroxidases across plant species [27], [29], [30], [31], and the primers also generated polymorphic DNA fingerprints in potato and *Miscanthus* (van der Linden, unpublished data). We tested twelve primers targeting both motifs with slight sequence variations to account for different codon usage and variation in the motifs, particularly at the 3′ end of the primers. The 3′ ends of PCR primers should ideally be non-degenerate to increase the specificity and efficiency of amplification. All primers produced polymorphic DNA fingerprints with 4 to 30 polymorphic markers per primer/restriction enzyme combination. A large fraction of the amplified bands was homologous to *Prx* sequences, but more so for FHDCFV-derived than for VSCADI-derived primers. The number of *Prx* Profiling markers can be increased further by testing additional restriction enzymes.

### Organization of *Prx* Profiling Markers in the Barley Genome

Most gene families in plant genomes seem to be organized in several large clusters of highly homologous genes, most likely resulting from various duplication events. Clustering of *Prx* genes has previously been observed in rice [27] and Arabidopsis [29]. The clustering of the markers mapped in this study adds to the evidence that many must indeed be targeting *Prx* genes. This clustering is in line with the fact that *Prx* genes belong to a gene family with evolutionary related tandemly repeated genes, or to allelic series [36].

The chromosomal distribution of FHDCFV and VSCADI-derived markers is very similar, and both typically mapped in the same clusters. We found 26 clusters with two or more *Prx* based markers. Fourteen clusters contained VSCADI markers as well as FHDCFV markers and/or DGH *Prx* markers (Table S5, Figures 1 and 2), One large cluster on 7H only contained thirteen VSCADI-derived PERO-markers, interspersed with some DGH *Prx* markers (Figures 1 and 2, Table S1). These findings suggest that VSCADI-derived bands correspond to *Prx* genes in spite of their lower *E* value compared to the FHDCFV-derived bands (Tables 2 and S2). Another cluster, on 2H, contained seven FHDCFV-derived PERO markers and three DGH markers. Such clusters of PERO-markers that are only based on the VSCADI or only on the FHDCFV motifs suggest that these clusters contain *Prx* genes that belong to a subfamily of highly similar genes.

Only few *Prx* genes had previously been mapped in barley, either as RFLP markers, viz. *Prx2* [13], *Prx7* [37], *Prx4* [38], or from *Prx*-like EST sequences. They were recently located on transcript maps of barley [13], [39], [40]. The fact that nine of the presently mapped PERO-marker clusters also contain previously mapped *Prx* markers indicates that the *Prx* Profiling markers indeed are *Prx* specific. In our study the largest clusters were found on linkage group 1H (1H_9.2), which includes 26 *Prx* Profiling markers, and on linkage group 2H (2H_15.1) with 18 *Prx* based markers.

The saturation approach followed here suggests that in barley there are about 40 of such clusters (see Figure 3). Also studies in other crops indicated that multigene families of plant *Prx*s tend to cluster within the genome [27], [29]. It would be of interest to compare whether the *Prx* clusters in barley coincide with *Prx* clusters on syntenic chromosome regions in other Gramineae, like rice, *Brachypodium distachyon* and maize, and whether they are also in those species associated with resistance to specialized biotroph pathogens. Such a comparison was beyond the scope of the present paper.

### *Prx* Profiling marker Sequences

We successfully demonstrated that a high proportion of the amplified DNA sequences generated by the *Prx* Profiling primers indeed have homology to known peroxidase genes (Table 2). Recently, a contig of three BAC clones covering nearly 300 Kb of barley cultivar Vada in the BIN 2H_15.1 was sequenced in our laboratory (unpublished data). A cluster of peroxidases previously identified in this region [13] was confirmed in the present study by 10 *Prx* Profiling markers mapping in the BIN 2H_15.1. Three of the genes annotated on the 300 Kb sequence are putative peroxidases. We searched the 300 Kb sequence for presence of PERO1 to PERO6 primer signature. PERO1 and PERO3 signatures did not detect anything, PERO4 and PERO6 specifically detected two of the three putative peroxidases, and PERO2 and PERO5 specifically detected all three putative peroxidases. Both conserved motifs were found in all three gene sequences, indicating that small variations at the DNA level determine whether the genes are recognized or not by PERO primers. This result provides further evidence of the specificity of the designed primers to detect peroxidase sequences and supports the idea that the primers targeting the VSCADI motif (PERO4, PERO5, PERO6) might be more specific than suggested by the sequences obtained in this study.

### Role of *Prx* Genes in Basal Resistance

Our study on a possible association between *Prx* genes and basal resistance was only possible because of the recent mapping of over 100 QTLs for basal resistance to several rust fungal species and to barley powdery mildew (Table S4). The barley mapping populations in which those QTLs were mapped were also used to build the dense integrated barley marker map used in the present study and two of those populations also to map the PERO-markers. This coherent and extensive data set indicates that *Prx* Profiling markers are significantly associated with QTLs for basal resistance. The association of *Prx* genes with resistance QTLs has been documented [2], [4], [7], [41], but their co-segregation had not been established until now. The most common way to identify a candidate gene that explains the QTL-effect on resistance is to look for map co-segregation between genes of interest and the QTLs for resistance [42]. Genes coding for recognition, signaling, and defense components have been identified with this strategy as candidates to explain resistance QTLs in several plant species [13], [14], [21], [22], [43], [44].

In a previous study [14], Jafary and associates found 13 co-localizations between QTLs for non-host resistance and DGH markers at less than 1cM; eight of the DGH markers were derived from *Prx* gene sequences. This suggests a higher association than one would expect to occur by chance of non-host resistance QTLs with *Prx* genes. These results were confirmed in the present study, and extended to basal resistance against barley powdery mildew. We found a significant (*P*<0.001) association between QTLs for basal host and non-host resistances and *Prx*-based markers. Moreover, all QTLs for resistance showed a significant (*P*<0.05) association with DGH markers only when the 32 *Prx*-based markers were included in this group.

The highly significant genetic association between *Prx* based markers and QTLs for resistance to different fungi found in this study is consistent with previous reports, supporting the idea that peroxidases are involved in plant defense reactions. We did not find such an association between resistance QTLs and markers based on random gene sequences and genomic sequences, nor between *Prx* based markers and QTLs for other agronomical traits than resistance (Table 4). Therefore, the clustering of *Prx* sequences at the same position as the known resistance QTLs makes *Prx* genes strong candidates for explaining the natural differences in resistance levels. Some *Prx* Profiling markers are associated with more than one resistance QTL, some effective to barley pathogens, others to pathogens to which barley is a marginal host. Regions harboring QTLs against different pathogen species could be explained by the presence of *Prx* gene clusters in which each *Prx* gene may have an effect against a different pathogen species. QTLs for partial resistance to leaf rust and QTLs for partial resistance to powdery mildew are significantly associated with *Prx* Profiling markers while no association was found between both types of resistances. The observed specificity of QTLs identified in different populations [14], [22], with different pathogen species [35] or even with different isolates of the same pathogen [33], resulted in more than 100 detected resistance QTLs in barley (Table S4, Table 4). If *Prx* genes indeed underlie many of the resistance QTLs, the observed abundance and specificity of resistance QTLs might be explained by the abundance of *Prx* genes and their varying allelic forms, each form having a narrow spectrum of effectiveness.

Definitive proof that peroxidases are involved in both types of basal resistance will nevertheless require transgenic complementation or *Prx*-gene specific gene silencing experiments.

Not all peroxidases may be involved in basal resistance, since they play a role in a broad range of physiological processes during the plant life cycle. Studies have suggested that peroxidases also play a role in germination, abiotic stresses, symbiosis, senescence and more [45]. Therefore, *Prx* Profiling may be useful for many other applications or traits of interest.

In our study 56% of the QTLs for resistance were linked to *Prx* Profiling markers (61% of QTLs for partial resistance to *P. hordei*, 61% for *B. graminis* and 47% for heterologous rusts). The QTLs not associated with *Prx* profiling markers may be associated with *Prx* genes not mapped in this study, or to other types of genes governing other defense mechanisms. Indeed not all resistance QTLs will be explained by *Prx* genes. Recently, the non-hypersensitive resistance gene *Lr34* has been cloned [46], which turned out to be an ABC transporter. Niks and Marcel [21] proposed that all kinds of genes involved in pathogen perception, signal transduction or defense are potential targets of effectors from would-be pathogens to suppress plant defenses. Our present study suggests that *Prx* genes may represent a substantial part of those targets.

## Materials and Methods

### Plant Material

The RIL populations used in the present study have been developed at Wageningen University (Wageningen, The Netherlands), and consist of 103 lines derived from a cross between L94 and Vada [32] and 152 lines derived from a cross between Vada and SusPtrit [35].

### Available Linkage Mapping Data

Recently, Aghnoum and associates [22] constructed a barley integrated map regrouping 6990 markers from 7 barley mapping populations, including L94×Vada and Vada×SusPtrit (“Barley, Integrated, Marcel 2009” at http://wheat.pw.usda.gov/). The most represented types of molecular markers on this integrated map are RFLP (20%), AFLP (20%), SSR (9%), DArT (19%) and TDM (23%) ( = 91% of all markers).

### Design of PCR Primers Targeting Peroxidase Sequences

For the design of degenerate primers that would recognize a broad spectrum of *Prx* genes, 105 protein sequences of peroxidase from barley were extracted from PeroxiBase [47] and aligned with ClustalX [48]. Two conserved amino acid motifs were identified in these sequences at about 150 base pairs from each other: FHDCFV and VSCADI. Twelve degenerate primers (named as PERO primers) were designed on those conserved motifs to amplify DNA towards the 5′end of the targeted *Prx* sequences (Table 5).

**Table 5:** Twelve specific degenerated primers were developed from two conserved motifs.

Primer name	Motif	Sequence (in 5′ - 3′order)
PERO1	FHDCFV	tsywyttccacgactgyttygt †
PERO2	FHDCFV	tsmgbmtsywyttccacgactg
PERO3	FHDCFV	ccyybvacraarcartcgtggaa
PERO4	VSCADI	sryngtstcvtgcgcngacat
PERO5	VSCADI	srbkatgtcngcrcabgagac
PERO6	VSCADI	srbkatgtcngcrcabgasac
PERO7	FHDCFV	tsmgbmtsywyttccaygaytg
PERO8	FHDCFV	tsywyttccacgaytgyttcgt
PERO9	FHDCFV	ttccacgaytgyttygtbvrrgg
PERO10	FHDCFV	ttccacgactgyttygtbvrggg
PERO11	FHDCFV	ccyybvacraarcartcgtgg
PERO12	VSCADI	sryngtstcvtgygcngacat

†Ambiguous DNA characters are represented using the standard notation recommended by the International Union of Biochemistry.

### Peroxidase Profiling Protocol

High DNA quality is an important prerequisite for Motif-directed Profiling. A combination of the classical CTAB-based protocol [49] complemented with additional purification steps (we added an additional CTAB step with a second incubation and repeated the isoamyl-chloroform step twice more) was applied to extract DNA from all samples. The extracted DNA was diluted to a concentration of 50 ng/µl before being processed.

*Prx* Profiling was developed according to the protocol described in [26] with some modifications. Restriction digestion and adapter ligation were performed in a single reaction by incubating 200 ng of DNA at 37°C for 3 h in the appropriate buffer and using high-concentration ligase (5U/µl). Amplification of *Prx*-specific fragments was performed in a single polymerase chain reaction with *Prx* primer and adapter primer as described in [50]. The PCR thermal profile was: 15 min at 95°C, 30 cycles at 95°C for 30 s for denaturing, 1 min 40 s at 60°C for annealing, 2 min at 72°C, and a final extension at 72°C for 20 min. Three different restriction enzymes (*MseI*, *AluI* and *RsaI*) were used in combination with the 12 *Prx*-specific degenerate primers. Examples of *Prx* –Profiling DNA fingerprints are given in Figure S1.

The PCR products were re-amplified using the adapter primer IRDye-labeled at Biolegio BV (Nijmegen, The Netherlands). The PCR reaction (5µL of 10× diluted PCR mixture, 1µL of 10× PCR buffer, 200µM dNTPs, 3 pmol of *Prx* primer, 0.6 pmol of IRD labeled adapter primer and 0.2 U of SuperTaq DNA in a final volume of 10 µL) was performed according to the following procedure: 3 min at 95°C followed by 35 cycles of 30 s at 95°C, 1.40 min at 60°C, and 2 min at 72°C; then a final extension step at 72°C for 20 min. The labeled PCR products were mixed with an equal volume (10µL) of formamide-loading buffer (98% formamide, 10mM EDTA pH 8.0 and 0.1% Bromo Phenol Blue) and an aliquot (0.8µL) was analyzed on a LI-COR 4300 DNA Analysis System (LI-COR Biosciences). The labeled PCR products were separated on 6% polyacrylamide gel as shown in Figure S1.

### Genetic Mapping

Polymorphic bands were scored for their presence/absence in the progeny. JoinMap 4 [51] was used to build the barley integrated map of 6990 markers [22] including the 168 scored PERO markers. The map also includes 32 *Prx*-based sequences that were mapped as CAPS, RFLP, SCAR or TDM markers [39], [52], [53].

### Homology of PERO Marker Sequences with *Prx* Genes

To determine the level of homology to known *Prx* genes of the DNA fragments amplified with the PERO primers designed in this study, 61 bands were excised from polyacrylamide gels after scanning with an Odyssey infrared imaging system (LI-COR Biosciences, Lincoln, NE, U.S.A.). Most of the bands isolated for primers PERO1 and PERO2 corresponded to markers mapped in L94×Vada or in Vada×SusPtrit populations while most of the bands isolated for primers PERO4, PERO5 and PERO6 were monomorphic in both populations. The bands were recovered by puncturing the polyacrylamide gel with a standard pipette tip, eluting DNA from the tip in TE for about 60 min at room temperature, and reamplified using similar conditions as the ones described for the exponential PCR protocol. PCR products were analyzed on a 1% agarose gel. Products appearing as clear and single bands were directly sequenced with both *Prx* and adapter primers using the BigDye Terminator kit on a LI-COR 4300 DNA Analysis System sequencer from Applied Biosystems (U.S.A.).

The quality of each sequence was determined by inspecting the ABI chromatogram with BioEdit sequence alignment editor (Copyright® 1997-2007 Tom Hall), and only good quality sequences were analyzed further. These sequences were compared to the peroxidase protein sequences from PeroxiBase (http://peroxibase.isb-sib.ch/) and against the protein sequences from NCBI database (http://www.ncbi.nlm.nih.gov/) using BLASTX [54] to determine their homology to known peroxidase sequences. The sequences were also compared with BLASTN against the DFCI Barley Gene Index database (http://compbio.dfci.harvard.edu/tgi/) to identify barley consensus EST sequences with highest homology to our sequences.

### Prediction of the Number of *Prx* Gene Clusters on the Barley Genome

A re-sampling procedure was followed to obtain an estimate of the total number of *Prx* clusters in the barley genome. This procedure is analogous to the approach that is applied in ecology for estimating the number of species or OTU's (operational taxonomic units) in a given geographic area or ecological niche [55].

We used the map positions of the PERO markers and other *Prx*-based markers to assess the number of observed clusters in our data. A *Prx* cluster was defined as a group of *Prx* based markers in which the largest distance between adjacent markers does not exceed a certain limit. This limit (the ‘gap distance’) was set to 5 cM. So the minimum distance between adjacent clusters is 5 cM. A special purpose program for the re-sampling procedure was written in C++; curve fitting was done with GenStat (VSN International Ltd., Oxford, UK). The re-sampling procedure ran as follows. From the total set of *Prx* markers a random sample, without replacement, was taken and these were arranged into clusters using the ‘gap size’ of 5 cM. For a given size of the sample this re-sampling was repeated 50,000 times and for each run the number of clusters in that sample was recorded. Finally, the average number of realized clusters over the 50,000 replicates was calculated.

We obtained a ‘saturation curve’ that levels off to an asymptotic value by carrying out this procedure for a range of sample sizes and plotting the average number of realized clusters against sample size,

### Association of *Prx* Profiling Markers with QTLs for Resistance

The map position of the 168 PERO markers and 32 other *Prx*-based sequences was compared with that of several QTLs, in order to test for independent distribution over the genome.

QTL positions in five of the populations composing the integrated map (Table S4) were used to test for association between *Prx* based markers and resistances to barley leaf rust [13], [14], [32], [33], to barley powdery mildew [22], [34], and to heterologous rusts ([14], [35] and unpublished QTLs by Jafary and Niks and Alemu and Niks). When resistance QTLs against a same pathogen species had overlapping confidence intervals on the integrated map, only one peak marker was considered. That peak marker from L94×Vada or Vada×SusPtrit was taken as the location of the QTL. In case the QTL to a same pathogen occurred in both populations, the peak marker with highest LOD value was taken as the position. If confidence intervals of QTLs for resistance to different heterologous rusts overlapped, they were still counted as different QTLs.

The co-segregations between *Prx* based and QTLs for basal host or non-host resistances were compared with the associations between *Prx* based markers and QTLs for agronomic traits, taken from GrainGenes database (http://wheat.pw.usda.gov/). We also tested for associations between QTLs for resistance and microsatellite markers derived from random expressed genes, viz. two sets of EST-SSRs composed of 244 GBM markers [56] and 34 scssr markers [57], and microsatellite markers derived from unspecified genomic sequences, viz. one set of Bmac+Bmag markers. Finally, we determined the association between QTLs for resistance and markers that are based on 97 DGHs (Defense Gene Homologues) [13], [22], and a subset of DGHs from which markers corresponding to *Prx* genes were omitted. The BIN system was used to realize chi-square tests to test the null hypothesis assuming independent distribution of BINs occupied with a *Prx* based markers and BINs occupied with a QTL peak marker or control molecular marker, as described previously [13]–[14].

## Supporting Information

Table S1 Position of Prx Profiling markers on Vada×SusPtrit and L94×Vada linkage maps and on the barley integrated map.
(0.05 MB XLS)

Table S2 Genetic position of PERO sequences on the barley integrated map and their homology to sequences from three different databases.
(0.05 MB XLS)

Table S3 35 PERO sequences in fasta format.
(0.01 MB TXT)

Table S4 Summary of QTLs conferring partial resistance to Puccinia hordei, to Blumeria graminis and to different heterologous Puccinia species.
(0.05 MB XLS)

Table S5 Clustering of the three types of Prx Profiling markers mapped in this study: PERO markers based on VSCADI motif, PERO markers based on FHDCFV motif and DGH Prx markers.
(0.03 MB DOC)

Figure S1 Examples of Prx Profiling fingerprints revealed by electrophoresis for three different enzyme-primer combinations on the L94×Vada mapping population. A: Alu.PERO1; B: Mse.PERO2; C: Rsa.PERO2.
(1.40 MB TIF)

Figure S2 The frequency distribution of Prx cluster sizes. Adjacent Prx based markers belong to the same cluster when their distance is at most 5 cM.
(0.06 MB TIF)
