# Supplementary material for: Peroxidase Profiling Reveals Genetic Linkage between Peroxidase Gene Clusters and Basal Host and Non-Host Resistance to Rusts and Mildew in Barley
Source: PLoS One. 2010 Aug 2;5(8):e10495. doi: 10.1371/journal.pone.0010495 (PMC2914007; doi:10.1371/journal.pone.0010495)
Supplement: Table S5 — Clustering of the three types of Prx Profiling markers mapped in this study: PERO markers based on VSCADI motif, PERO markers based on FHDCFV motif and DGH Prx markers. (0.03 MB DOC) [file pone.0010495.s005.doc]

| clusters made up of VSCADI based markers | 4 | 15.38% |
| --- | --- | --- |
| clusters made up of FHDCFV based markers | 4 | 15.38% |
| clusters made up of DGH *Prx* | 1 | 3.85% |
| clusters made up of both *Prx* profiling markers | 8 | 30.77% |
| clusters made up of FHDCFV motif and DGH *Prx* markers | 3 | 11.54% |
| clusters made up of VSCADI motif and DGH *Prx* markers | 2 | 7.69% |
| clusters made up of the three types of markers | 4 | 15.38% |
| Total of cluster with ≥ 2 *Prx* | 26 |  |

**Table S5.** Clustering of the three types of *Prx* Profiling markers mapped in this study: PERO markers based on VSCADI motif, PERO markers based on FHDCFV motif and DGH *Prx* markers.
