# Supplementary figures and images for: Peroxidase Profiling Reveals Genetic Linkage between Peroxidase Gene Clusters and Basal Host and Non-Host Resistance to Rusts and Mildew in Barley
Source: PLoS One. 2010 Aug 2;5(8):e10495. doi: 10.1371/journal.pone.0010495 (PMC2914007; doi:10.1371/journal.pone.0010495)

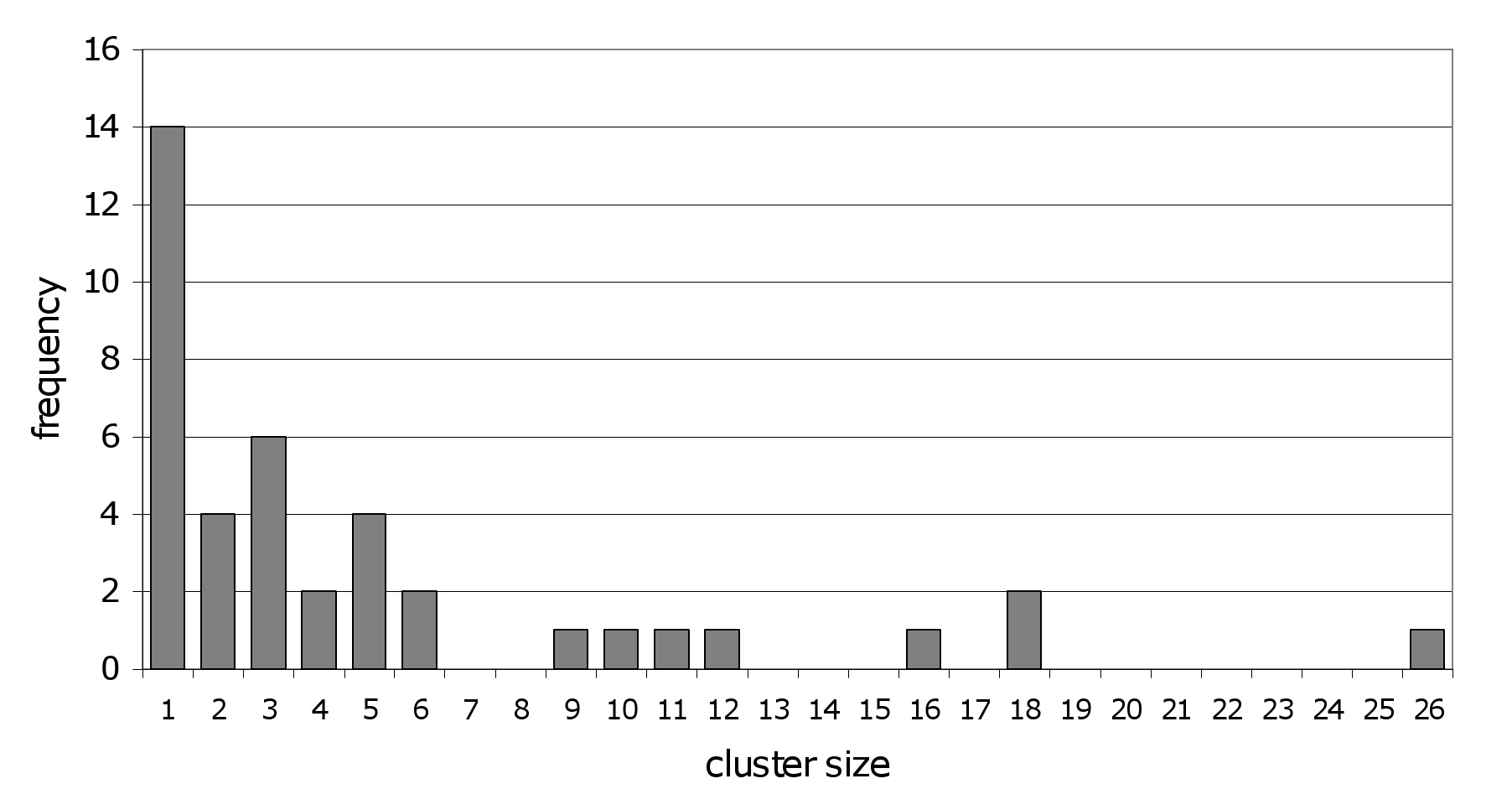

Supplement: Figure S2 — The frequency distribution of Prx cluster sizes. Adjacent Prx based markers belong to the same cluster when their distance is at most 5 cM. (0.06 MB TIF) [file pone.0010495.s007.tif]
